# Examining the Feasibility, Acceptability, and Effectiveness of Remote Training on Community-Based Participatory Research: Single-Arm Pre-Post Pilot Study

**DOI:** 10.2196/48707

**Published:** 2024-03-01

**Authors:** Karen Fortuna, Andrew Bohm, Stephanie Lebby, Kisha Holden, Branka Agic, Theodore D Cosco, Robert Walker

**Affiliations:** 1 Department of Psychiatry Geisel School of Medicine at Dartmouth Lebanon, NH United States; 2 The Dartmouth Institute for Health Policy and Clinical Practice Geisle School of Medicine Darmouth College Hanover, NH United States; 3 College of Nursing and Health Sciences The University of Vermont Burlington, VT United States; 4 Department of Psychiatry and Behavioral Sciences Morehouse School of Medicine Atlanta, GA United States; 5 Centre for Addiction and Mental Health University of Toronto Toronto, ON Canada; 6 School of Public Policy, Simon Fraser University Vancouver, BC Canada; 7 Oxford Institute of Population Ageing, University of Oxford Oxford United Kingdom; 8 Collaborative Design for Recovery and Health Nashua, NH United States

**Keywords:** community-based participatory research, CBPR, peer support, health literacy, remote training, community-based, user, mental health

## Abstract

**Background:**

Over the past decade, a growing body of scientific evidence has demonstrated that community engagement in research leads to more relevant research, enhances the uptake of research findings, and improves clinical outcomes. Despite the increasing need for the integration of community engagement methodologies into the scientific inquiry, doctoral and master's level competencies in the field of psychiatry often lack dedicated training or coursework on community engagement methodologies.

**Objective:**

A total of 13 service users, peer support specialists, caregivers of people with mental health challenges, and scientists (with specialties ranging from basic science to implementation science) aged 18 and older participated in remote training on community-based participatory research. Data were collected at baseline, 2 days, and 3 months.

**Methods:**

A total of 13 service users, peer support specialists, caregivers of people with mental health challenges, and scientists (with specialties ranging from basic science to implementation science) aged 18 and older participated in remote training on community-based participatory research. Data were collected at baseline, 2 days, and 3 months.

**Results:**

The pilot study demonstrated that a 3-month remote training on community-based participatory research (“Partnership Academy”) was deemed feasible and acceptable by service users, peer support specialists, caregivers of people with mental health challenges, and scientists. Improvements were found in research engagement and the quality of partnership. A marked increase in distrust in the medical system was also found. Groups submitted 4 grant applications and published 1 peer-reviewed journal at a 3-month follow-up.

**Conclusions:**

This pre- and postpilot study demonstrated it is possible to train groups of service users, peer support specialists, caregivers of people with mental health challenges, and scientists in community-based participatory research. These findings provide preliminary evidence that a 3-month remote training on community-based participatory research (“Partnership Academy”) is feasible, acceptable, and potentially associated with improvements in research engagement as well as the quality of partnership and output, such as manuscripts and grant applications.

## Introduction

Over the past decade, growing scientific evidence shows that community engagement in research produces more relevant research, increases uptake of research findings, and improves clinical outcomes [1-6]. Community engagement is defined as “a process of working collaboratively with groups of people who are affiliated by geographic proximity, special interests, or similar situations, with respect to issues affecting their well-being” [1]. Patient-centered outcomes research (PCOR) is intended to improve community engagement in research, yet it often leaves partners feeling overburdened and disenfranchised, leading to premature disengagement from PCOR [6].

Community engagement is crucial to addressing health disparities through the inclusion of historically underrepresented and disadvantaged populations in mental health research. Service users of the mental health system are primarily individuals from low-income groups who have disabilities, multiple chronic health conditions, and low health literacy. This population commonly disengages from research due to mistrust rooted in historical traumatic experiences in the mental health system, which in turn leads to the lack of representation in PCOR. As such, significant investment in the science of community engagement is needed to improve community engagement in PCOR [7].

Despite the need for the integration of community engagement methodologies into the scientific inquiry, doctoral and master’s level competencies in the field of psychiatry commonly do not include dedicated training or coursework on community engagement methodologies [2]. Without appropriate training or research experience, attempts to facilitate community engagement in research are often ineffective and burdensome, leaving partners feeling disengaged [3]. The purpose of this study was to assess the feasibility, acceptability, and preliminary effectiveness of remote training on community-based participatory research—“Partnership Academy.”

## Methods

### Procedures

The authors participated in and are members of the Early Mortality in People with a Diagnosis of a Serious Mental Illness (SMI) roundtable convened remotely on May 24 and 26, 2022. The roundtable was a diverse, interdisciplinary partnership collaborative composed of individuals with lived experience of mental health or substance misuse, peer support specialists, recovery coaches, parents and caregivers of people with SMIs, researchers and clinician-scientists with and without lived experience, policy makers, and representatives from patient-led organizations committed to addressing the health disparity in early mortality among people with SMIs through patient-centered research. To date, no such collaboration of partners exists. The roundtable aimed to advance the understanding of fundamental patterns and interactions among and between environmental, behavioral, cultural, neurobiological, psychological, and biopsychosocial mechanisms on health and health behavior relevant to early mortality in people with SMIs.

Roundtable members were selected by reviewing the published literature on early mortality and SMIs. KF and RW conducted a Google Scholar search using variations of the following search terms: “early mortality” and “serious mental illness.” Next, these authors (KF and RW) emailed authors included in the identified prereviewed manuscripts. Identified members recommended additional members through a snowball sampling framework. Patient partners were identified through direct email to partners of the Collaborative Design for Recovery and Health, which is an international group of patients, clinicians, peer support specialists, caregivers, scientists with and without lived experiences, policy makers, and payer systems led by KF and RW. The Collaborative partnered with different community groups from vulnerable populations across the intersectionality of disability and race to coproduce solutions to address community-identified challenges.

Attendants of the Early Mortality in People with a Diagnosis of a Serious Mental Illness roundtable were also given the option to complete surveys before day 1, after day 2, and 3 months after the roundtable. The surveys were used to assess the impact of the training on partners. KF provided participants with a detailed description of the study protocol if they were interested, and a survey link was emailed to individuals with the digital informed consent form. Participants clicked “I agree” on the informed consent form to participate and completed the web-based baseline survey.

The roundtable convening used a remote community meeting method, adhering to the Peer and Academic Partnership model of community engagement [8]. The Peer and Academic Partnership is based on the Center for Disease Control and Prevention’s principles of community engagement (2011) [9], as follows: (1) develop a clear understanding of the purpose, goal, and population involved in community change; (2) become knowledgeable about all aspects of the community; (3) interact and establish relationships with the community; (4) encourage community self-determination; (5) partner with the community; (6) respect community diversity and culture; (7) activate community assets and develop capacity; (8) maintain flexibility; and (9) commit to long-term collaboration. Although the project team initially considered convening in person, the rapid rise in remote meetings due to COVID-19 has highlighted the benefits of remote convening, especially for the early mortality community, given its international representation.

The roundtable members included people across the United States, United Kingdom, Canada, Europe, Africa, Australia, Asia, and the Netherlands. With such a geographically dispersed community, an in-person convening was not feasible for all partners and would have involved disproportionate travel expenses. To ensure a productive remote meeting, the project team offered to train members to use the virtual platform before the roundtable and planned a rehearsal to work through any last-minute challenges with the meeting platform. Further, to facilitate equitable access to engagement, members were encouraged to call in and not use video if their available technology did not allow for video.

The roundtable convened over two 5-hour days across 1 week in May 2022, structured as a summit with several remote meeting sessions. KF and RW facilitated the summit, set the tone, provided participation guidelines, and kept discussions focused and oriented to the goals of the roundtable. The roundtable used a Delphi method to achieve a consensus on the research agenda. The Delphi method is an empirically supported process used to attain consensus within an expert group [10]. Roundtable members responded to several rounds of PCOR research agenda development. After each round, their responses were aggregated and shared with the group until a consensus was achieved.

Patients made up at least 60% of meeting participants, and all verbal and written materials for the convening meeting were designed with consideration for potential cognitive and intellectual needs, following principles of design for people with SMIs (eg, information presented at fourth-grade level and single structure sentences). Further, all interactions were based on adult learning techniques designed to reduce cognitive effort and promote engagement among all members. For example, KF encouraged the roundtable to share their respective perspectives on early mortality (personal or research-related perspectives) to promote discussions (ie, experiential learning theory), and RW used a round-robin technique to encourage all members to share their ideas, built-in breaks, and energizers into sessions to keep roundtable members engaged, positive, and productive. The community engagement techniques used each day are delineated in the following sections.

#### Convening Meeting Day 1: Setting the Stage and Story With a Gap

The first session began with a welcome and an opportunity for introductions, followed by a session on the historical literature review of early mortality among people with SMIs. This was followed by a large group discussion intended to identify gaps in our understanding of early mortality among people with SMIs. Next, we presented a Story with a Gap to elicit gaps in the extant research. The Story with a Gap technique includes 2 contrasting pictures of “before” and “after” situations [11]. Following this technique, roundtable members identified the steps and resources needed to move from the “before” to the “after” situation. In conclusion, opportunities to lead committees to work toward tasks identified in the strategic planning process were formed. Next, each member evaluated and ranked their foci for future research, using anonymous polling videoconferencing from the first session to select the 3 highest impact areas within the bounds of financial, time, and other constraints.

#### Convening Meeting Day 2: Multiple Rounds of Delphi and Consensus

The first session of day 2 began with a draft PCOR research agenda based on discussions from day 1. The PCOR research agenda included, at a minimum, strategies to address gaps in research efforts. All partners commented on the PCOR research agenda and first proposed recommendations publicly in an open forum and, second, proposed additional recommendations anonymously using a Qualtrics web-based survey. This iterative process occurred until a consensus was reached. During day 2 sessions, RW implemented techniques to promote conversation. He used brainstorming, “Go Wild” prompts (ie, asking roundtable members to talk about ideas that begin with “wouldn’t it be good if...”), and reverse brainstorming (ie, considering the reverse of problems) to generate creative, thoughtful, and innovative ideas regarding early mortality PCOR. Then, in the Reality Check session, RW used multivoting, ranking, and problem-solving methods to help the roundtable make decisions about which ideas were most feasible and impactful and how to overcome barriers to their implementation.

### Study Design and Participants

The study used a single-arm pre- and postdesign approach to assess the impact of training partners from diverse groups designed to facilitate community-engaged research. Participants (N=13) included service users, peer support specialists, caregivers of people with mental health challenges, and scientists (basic science to implementation scientists).

### Ethical Considerations

This study was approved by the Dartmouth Health institutional review board (STUDY02001532).

### Instruments

#### Quality of Partnership

The quality of PCOR was assessed using the Quality of Patient-Centered Outcomes Research Partnerships Instrument (QPCOR) [12]. The QPCOR contains the following domains: (1) purpose, goal, and population; (2) respect (respect community diversity and culture); (3) inclusion (activate community assets); (4) colearning (develop capacity); (5) become knowledgeable about the community; (6) self-determination; (7) shared decision-making (partner with the community); (8) perceived support (interact and establish relationships with the community); (9) flexibility; and (10) sustainability (commitment to long-term collaboration). The QPCOR uses a 10-point Likert scale. Items with a score of 60 or lower indicate the need for improvement and should be addressed. Higher scores indicate higher levels of partnership.

#### Engagement

Engagement was measured using The Research Engagement Survey Tool (REST). The REST is a 9-item scale that evaluates the level of nonacademic partner engagement among research partners. Example items include “The focus is on problems important to the community” and “All partners assist in establishing roles and related responsibilities for the partnership.”

The REST is measured on 2 Likert-type scales (for quantity and quality). The response options for the quantity scale were “never,” “rarely,” “sometimes,” “often,” “always,” and “not applicable.” The response options for the quality scale were “poor,” “fair,” “good,” “very good,” “excellent,” and “not applicable.” Responses were coded in order from 1 to 5 for both scales, with higher scores indicating higher engagement; not applicable options were coded as missing. For the REST, mean scores were calculated overall for both quality and quantity scales. The overall mean scores for both scales were created by averaging the mean scores so that each response is weighted equally regardless of the number of items.

#### Distrust in the Medical System

Distrust in the medical system was measured using the Health Care System Distrust Scale [13]. The Health Care System Distrust Scale contains 10 items and is measured on a Likert scale. Example prompts include “Medical experiments can be done on me without my knowing about it” and “My medical records are kept private.” Scores on the Health Care System Distrust Scale range from 12 to 46 with a possible range from 10 to 50. The score is the sum of 10 questions from the Health Care System Distrust Scale after reversing 2 positively framed items. The possible range is from 10 to 50.

#### Effectiveness

Effectiveness was assessed by collecting data at the 3-month mark, including progress toward grant submissions, submitted manuscripts, and changes in research knowledge.

### Data Analysis

Descriptive statistics were conducted to describe the demographic characteristics of the study sample. A paired-sample *t* test was conducted to assess the difference between baseline, day-2, and 3-month scores for statistical significance. Participants served as their own controls from pre- to posttest. Descriptive statistics and analyses were computed using STATA (version 13.1; StataCorp). The statistical models used to analyze the data accommodate missing data, assuming that they are missing at random.

## Results

Demographically, the population of this feasibility study was predominantly female (n=8, 62%), White (n=10, 77%), and educated at or above a master’s level (n=8, 62%). Study participants represented a wide range of adult age groups with the plurality being in the age category of 45-55 years, and there was a wide range of partners represented (Table 1).

For all 3 survey tools used in this study (Healthcare System Distrust Scale, REST, and Quality of Patient-Centered Outcomes Research Measurement tool), there was a marked but not statistically significant increase from pre- to posttest. The Distrust Scale and REST (5-point scales) both increased 0.03 units (*P*=.75 and *P*=.85, respectively), representing increased distrust and research engagement in the postmeeting survey. There was also a marked increase of 6.86 units in the Quality of Patient-Centered Outcomes Research Measurement tool (*P*=.20; Table 2).

When evaluating individual questions, some participants had a more significant degree of change in the postmeeting setting compared to others. In particular, a question regarding providers hiding medical mistakes showed significantly more agreement (mean 3.38, SD 1.04 vs mean 2.69, SD 1.03; *P*=.04; Hedges *g*=0.65), and participants indicated significantly more comfort in engaging with research study team members (mean 91.00, SD 15.36 vs mean 77.15, SD 27.36; *P*=.049; Hedges *g*=0.60) in the postmeeting survey. Many other questions demonstrated meaningful but not significant increases with a universal increase in survey responses for the Quality in Patient-Centered Outcomes Research Measurement tool (ie, change range per question: minimum +3.54; maximum +14.23; Hedges *g* range: 0.13-0.60).

The Health Care System Distrust Scale included some items that presented an increase on the scale and some that presented a decrease. The most notable increase in distrust was hiding medical errors, as previously mentioned. The most notable decrease (represented by increased trust in the medical system) was related to the health care system putting medical needs as a priority over all other issues during care (mean 2.85, SD 0.99 vs mean 3.31, SD 1.11; *P*=.14; Hedges *g*=–0.43). In the REST tool, there was a minimal change for most questions, with most questions exhibiting a ceiling effect and being near 5 (highest score) in both the pre- and postsetting (Table 3). There were no statistically significant differences in mean scores pre- and postintervention for any domain covered by the REST tool.

**Table 1:** Sociodemographic characteristics of individuals involved in the research academy. Partner categories are not mutually exclusive, and participants may be represented in multiple categories (N=13).

Characteristics		Values, n (%)
**Gender**		
	Female	8 (62)
	Male	5 (39)
**Race**		
	Asian-Eastern	0 (0)
	Asian-Indian	0 (0)
	Black/African American	2 (15)
	Hispanic	0 (0)
	Multiple races	1 (8)
	Native American	0 (0)
	White	10 (77)
**Age (years)**		
	18-24	0 (0)
	25-34	1 (8)
	35-44	2 (15)
	45-54	4 (31)
	55-64	3 (23)
	65-74	3 (23)
**Highest level of education**		
	Graduate degree or above	8 (62)
	Bachelor’s degree	2 (15)
	High school degree	1 (8)
	Other	2 (15)
**Partner role**		
	Caregiver of person(s) with SMI^a^	4 (31)
	Service user	4 (31)
	Peer support specialist	6 (45)
	Scientist	6 (45)
	Other	1 (8)

^a^SMI: serious mental illness.

**Table 2:** Pre- and postchanges on outcomes of interest.

Outcome of interest	Prechange, mean (SD)	Postchange, mean (SD)	Mean difference (SD)	*P* value	Effect size (95% CI)^a^
Health care system distrust	3.08 (0.55)	3.12 (0.50)	0.03 (0.34)	.75	0.06 (–0.69 to 0.80)
REST^b^	4.56 (0.60)	4.60 (0.58)	0.03 (0.60)	.85	0.06 (–0.69 to 0.80)
QPCOR^c^	79.01 (22.80)	85.87 (22.59)	6.86 (18.28)	.20	0.29 (–0.46 to 1.04)

^a^Hedges *g* was used to calculate effect sizes (due to the small sample size).

^b^REST: Research Engagement Survey Tool.

^c^QPCOR: Quality of Patient-Centered Outcomes Research.

**Table 3:** Pre- and postchanges by individual questions.

Characteristics		Prechange mean (SD)	Postchange mean (SD)	Mean difference SD	*P* value		Effect size (95% CI)^a^	
**Healthcare System Distrust Scale^b^**								
	Medical experiments can be done on me without my knowing about it.	2.23 (1.36)	2.23 (1.01)	0 (0)	1.000		0 (–0.74 to 0.74)	
	My medical records are kept private.	4.23 (0.83)	3.92 (0.76)	–0.31 (0.63)	0.104		–0.37 (–1.12 to 0.38)	
	People die every day because of mistakes by the health care system.	3.85 (1.07)	3.69 (0.85)	–0.15 (0.90)	0.55		–0.15 (–0.90 to 0.59)	
	When they take my blood, they do tests they don’t tell me about.	2.62 (1.19)	2.31 (0.95)	–0.31 (1.18)	0.37		–0.28 (–1.02 to 0.47)	
	If a mistake were made in my health care, the health care system would try to hide it from me.	2.69 (1.03)	3.38 (1.04)	0.69 (1.11)	0.040		0.65 (–0.12 to 1.41)	
	People can get access to my medical records without my approval.	2.54 (0.97)	2.85 (1.07)	0.31 (1.25)	0.393		0.29 (–0.45 to 1.04)	
	The health care system cares more about holding costs down than it does about doing what is needed for my health.	3.15 (0.99)	3.38 (1.19)	0.23 (1.30)	0.535		0.20 (–0.54 to 0.95)	
	I receive high-quality medical care from the health care system.	3.62 (1.12)	3.77 (0.97)	0.15 (0.99)	0.585		0.14 (–0.60 to 0.89)	
	The health care system puts my medical needs above all other considerations when treating my medical problems.	3.31 (1.11)	2.85 (0.99)	–0.46 (1.05)	0.139		–0.43 (–1.18 to 0.33)	
	Some medicines have things in them that they don't tell you about.	2.62 (1.33)	2.77 (1.17)	0.15 (1.21)	0.656		0.12 (–0.63 to 0.86)	
**Research Engagement Survey Tool^b^**								
	The focus is on problems important to the community.	4.69 (0.63)	4.76 (0.60)	0.08 (0.95)	0.776		0.12 (–0.87)	
	All partners assist in establishing roles and related responsibilities for the partnership.	4.53 (0.77)	4.53 (0.88)	0 (1.08)	1.000		0 (–0.74 to 0.74)	
	Community-engaged activities are continued until the goals (as agreed upon by all partners) are achieved.	4.38 (0.87)	4.38 (0.77)	0 (0.71)	1.000		0 (–0.74 to 0.74)	
	The partnership adds value to the work of all partners.	4.69 (0.48)	4.61 (0.77)	–0.08 (0.76)	0.721		–0.12 (–0.86 to 0.63)	
	The team builds on strengths and resources within the community or patient population.	4.62 (0.51)	4.62 (0.65)	0 (0.71)	1.000		0 (–0.74 to 0.74)	
	All partners’ ideas are treated with openness and respect.	4.54 (0.66)	4.69 (0.63)	0.15 (0.80)	0.502		0.23 (–0.52 to 0.98)	
	All partners agree on the timeline for making shared decisions about the project.	4.46 (0.78)	4.54 (0.78)	0.08 (0.49)	0.585		0.10 (–0.65 to 0.84)	
	The partnership’s processes support trust among all partners.	4.62 (0.65)	4.69 (0.63)	0.07 (0.95)	0.776		0.12 (–0.63 to 0.86)	
	Mutual respect exists among all partners.	4.54 (0.66)	4.54 (0.88)	0 (1.00)	1.000		0 (–0.74 to 0.74)	
**Quality of Patient-Centered Outcomes Research^c^**								
	I had a clear understanding of the purpose of the study.	78.6 (26.27)	82.92 (26.44)	4.31 (15.00)	0.321		0.16 (–0.59 to 0.90)	
	I felt listened to.	76.54 (26.98)	85.62 (28.81)	9.08 (26.90)	0.247		0.31 (–0.44 to 1.06)	
	I felt prepared to be an equal partner in the research study.	80.77 (24.17)	84.62 (21.43)	3.85 (16.26)	0.411		0.16 (–0.58 to 0.91)	
	Researchers were knowledgeable about people like me or were willing to learn about people like me.	81.77 (21.60)	86.69 (20.38)	4.92 (24.28)	0.479		0.23 (–0.52 to 0.97)	
	I believe that I had choices in how I could be part of the research study.	80.38 (24.10)	83.93 (28.50)	3.54 (28.98)	0.668		0.13 (–0.62 to 0.87)	
	I feel prepared to be an equal partner in the research study.	80.92 (25.28)	84.62 (26.26)	3.69 (21.65)	0.550		0.14 (–0.60 to 0.88)	
	I feel accepted by all members of the research study team.	80.31 (24.30)	85.85 (26.87)	5.54 (24.65)	0.434		0.21 (–0.54 to 0.95)	
	Researchers used language that was consistent with my values and culture.	77.69 (27.64)	84.77 (26.31)	7.08 (24.52)	0.319		0.25 (–0.50 to 1.00)	
	Both community members and researchers are thinking of ways we can continue to work together in the future.	75.00 (27.49)	89.23 (18.58)	14.23 (29.62)	0.109		0.59 (–0.18 to 1.34)	
	I felt comfortable engaging with the members of the research study team.	77.15 (27.36)	91.00 (15.36)	13.85 (22.85)	0.049		0.60 (–0.17 to 1.36)	
	I felt my views were incorporated into the research study.	79.92 (27.89)	85.31 (26.07)	5.38 (18.71)	0.320		0.19 (–0.56 to 0.94)	

^a^Hedges *g* was used to calculate effect size (due to the small sample size).

^b^On a 5-point Likert scale.

^c^On a 10-point scale.

## Discussion

### Principal Findings

The purpose of this study was to assess the feasibility, acceptability, and preliminary effectiveness of remote training on community-based participatory research. The Partnership Academy was found to be feasible and acceptable. Improvements were found in research engagement and the quality of the partnership. A marked increase in distrust in the medical system was also found. Three months after the Partnership Academy training, the trainees submitted 4 grant applications and published 1 peer reviewed research article.

Feasibility and acceptability by service users, peer support specialists, caregivers of people with mental health challenges, and scientists were demonstrated through their capacity to attend and participate in the Partnership Academy. With the geographically dispersed community of the Partnership Academy, an in-person meeting might not be feasible for all partners and could invoke disproportionate travel expenses. Remote training allowed partners from all parts of the United States to meet and work together while avoiding travel, accommodation, and facility rental expenses. Further, all aspects of the remote training were aligned with the Americans with Disability Act requirements. For example, patients with cognitive impairments may have difficulty using Zoom due to challenges related to motion sensitivity. As such, there was no requirement to use the video feature.

The Partnership Academy was found to be potentially effective in promoting research engagement. Greater alignment of partner priorities and researchers’ objectives facilitates greater engagement in all parts of the research process, from study conceptualization to knowledge mobilization, ultimately increasing the likelihood of an intervention’s success [14]. This shift goes beyond a paradigm where research functions as a one-way conversation, to one in which active community participation has facilitated and enabled greater integration and engagement of partners and researchers alike [15]. The model of the Partnership Academy exemplifies these concepts and practically implements their use, providing evidence for the potential effectiveness of this approach in prospective research projects. Other trainings are available, such as the Community-based Participatory Research Academy [16] and Patient-Centered Outcomes Research Institute’s Research Fundamentals: Preparing You to Successfully Contribute to Research; however, they have not been designed for the unique needs of people with SMIs.

A marked increase in distrust in the medical system was also found. There are a few possible explanations for an increase in distrust in a medical system, in particular concerns for medical errors. First, it is possible that increased awareness from the conversations during the training led to further entrenchment in previously held beliefs. Second, it is possible that during the 2-day training, the roundtable participants heard not only more negative stories about the medical errors but also negative stories from fellow roundtable members who were deemed “credible” as scientists, expressing their concern with medical errors. Integrating qualitative data collection in future roundtable events may elicit new knowledge regarding perceptions of mistrust among participants.

### Limitations

Due to the nature of the study and the study design, there are inherent limitations. There are additional limitations surrounding the study design and analysis of data. Due to the small sample size, we could not stratify participants by demographic characteristics or differing experiences with different SMIs. It is unknown if different participants with different diagnoses, or researchers who engaged in diagnosis-specific research had different program evaluations. Additionally, due to the Likert-scale measurements, understanding the true magnitude of the effect is limited to categorical shifts and not continuous measurement changes. Moreover, Likert questions open the study up to potential acquiescence bias due to participants’ potential to overly agree with statements (in comparison to their actual feelings). Due to the sampling methodology used, those selected for participation in the roundtable were those who were actively engaged in the health care system or research, and these findings may not apply to the population with SMI. Lastly, less than 25% of the study population represents racial minorities or those with lower levels of education. Future studies should make an effort to recruit a more diverse group of participants.

### Conclusions

This pre- and postpilot study demonstrated the possibility of training groups of service users, peer support specialists, caregivers of people with mental health challenges, and scientists in community-based participatory research. These findings provide preliminary evidence that a 3-month remote training on community-based participatory research (“Partnership Academy”) is feasible and acceptable and potentially associated with improvements in research engagement as well as the quality of partnership and output, including coproduced grant applications and peer-reviewed manuscripts.

Addressing the multifaceted health needs as well as the mental and behavioral health needs of diverse individuals, families, and communities in the United States is a complex issue that warrants attention from clinicians, researchers, scientists, public health professionals, and policy makers. The use of a community-based participatory framework supports the notion of implementing innovative approaches to help address health and mental health disparities. Moreover, our study reinforces key tenets of values delineated through inventive collaborations and partnerships that may be promising. In particular, our engagement and training efforts suggest the significance of (1) building trust and relationships, (2) establishing a shared purpose and vision for the achievement of goals, (3) engendering transparency and effective communication, and (4) performing continuous quality improvement or process and outcome evaluation where appropriate. Advancing health equity requires multidimensional, multisectoral, and interdisciplinary approaches to adequately address the needs of ethically and culturally diverse populations.

## Abbreviations

PCOR: patient-centered outcomes research
QPCOR: Quality of Patient-Centered Outcomes Research Partnerships Instrument
REST: Research Engagement Survey Tool
SMI: serious mental illness
